# HANDCYCLING WITH CONCURRENT LOWER BODY LOW-FREQUENCY ELECTROMYOSTIMULATION SIGNIFICANTLY INCREASES ACUTE OXYGEN UPTAKE IN ELITE WHEELCHAIR BASKETBALL PLAYERS: AN ACUTE CROSSOVER TRIAL

**DOI:** 10.2340/jrm.v56.40028

**Published:** 2024-06-08

**Authors:** Ludwig RAPPELT, Steffen HELD, Florian MICKE, Tim WIEDENMANN, Jan-Philip DEUTSCH, Heinz KLEINÖDER, Lars DONATH

**Affiliations:** 1 Department of Intervention Research in Exercise Training, German Sport University Cologne, Cologne, Germany; 2 Department of Movement and Training Science, University of Wuppertal, Wuppertal, Germany; 3 Department of Sport and Management, IST University of Applied Sciences, Düsseldorf, Germany

**Keywords:** cardiorespiratory fitness, EMS, electrical muscle stimulation, endurance, SCI, spinal cord injury

## Abstract

**Objective:**

Wheelchair basketball (WCB) demands high-intensity training due to its intermittent nature. However, acute oxygen uptake (V̇O_2_) in handcycling is restricted. Combining handcycling with low-frequency electromyostimulation (LF-EMS) may enhance V̇O_2_ in elite WBC athletes.

**Design:**

Randomized crossover trail.

**Subjects:**

Twelve German national team WCB players (age: 25.6 [5.6] years, height: 1.75 [0.16] m, mass: 74.0 [21.7] kg, classification: 2.92 [1.26]).

**Method:**

Participants underwent 2×5 min of handcycling (60 rpm, ¾ bodyweight resistance in watts) (HANDCYCLE) and 2×5 min of handcycling with concurrent LF-EMS (EMS_HANDCYCLE). LF-EMS (4Hz, 350µs, continuous stimulation) targeted gluteal, quadriceps, and calf muscles, adjusted to individual pain thresholds (buttocks: 69.5 [22.3] mA, thighs: 66.8 [20.0] mA, calves: 68.9 [31.5] mA).

**Results:**

Significant mode-dependent differences between HANDCYCLE and EMS_HANDCYCLE were found in V̇O_2_ (17.60 [3.57] vs 19.23 [4.37] ml min^-1^ kg^-1^, *p* = 0.001) and oxygen pulse (16.69 [4.51] vs 18.41 [5.17] ml, *p* = 0.002). ΔLactate was significantly lower in HANDCYCLE (0.04 [0.28] vs 0.31 [0.26] mmol l^-1^). Although perceived effort did not differ (*p* = 0.293), discomfort was rated lower in HANDCYCLE (1.44 [1.28] vs 3.94 [2.14], *p* = 0.002).

**Conclusion:**

LF-EMS applied to the lower extremities increases oxygen demand during submaximal handcycling. Thus, longitudinal application of LF-EMS should be investigated as a potential training stimulus to improve aerobic capacity in wheelchair athletes.

LAY ABSTRACT

In wheelchair basketball, players have to undergo high-intensity training, but their oxygen uptake during handcycling is limited. We explored a solution: combining handcycling with low-frequency electromyostimulation. Twelve elite German wheelchair basketball players participated in our study. They performed handcycling alone and with electromyostimulation targeting specific muscles of the leg. Results showed that adding electromyostimulation significantly increased oxygen uptake during handcycling compared with the standalone exercise. Additionally, discomfort was only slightly lower with electromyostimulation, making it a promising technique for enhancing cardiovascular training in wheelchair athletes. This approach could potentially improve aerobic capacity, benefiting the overall performance of wheelchair athletes in clinical practice.

Wheelchair basketball (WCB), a popular sport within the Paralympics, is predominantly aerobic (1), but interspersed by short bouts of high-intensity activities (e.g., wheelchair maneuvering, ball handling) (2, 3). Thus, it has been suggested that training for WBC players should predominantly be of high intensity (1). In this context, effective high-intensity training is thought to require prolonged periods at training intensities corresponding to a high percentage of maximal oxygen uptake (V̇O_2_max) (4). However, as acute oxygen uptake (V̇O_2_) is influenced by muscle mass involvement (5), upper-body-limited exercises (e.g., handcycling) may result in lower V̇O_2_ compared with lower-body exercises (e.g., traditional cycling ergometry) (6). Therefore, arm exercise might be less effective for developing high levels of V̇O_2_max, presenting a particular challenge for athletes in wheelchair sports.

Underlying physiological adaptations for improvements in V̇O_2_max are generally either attributed to peripheral parameters affecting the arteriovenous oxygen difference or central factors improving the stroke volume at a given heart rate (7). While the peripheral factors are mainly affected by increased mitochondrial volume density and capacity or capillarization, the central adaptations are attributed to an increase in the ejection fraction induced by a reduced cardiac afterload and an increased end-diastolic volume (7). Given that electromyostimulation (EMS) was previously found to increase venous blood flow (8, 9) and thus may lead to an increased end-diastolic volume, EMS may be a potential complementary training method predominantly for wheelchair sports athletes in order to improve systemic function. Moreover, EMS of lower limb muscles can prevent muscular atrophy in spinal cord injury patients (SCI) (10), enhance haemodynamic and metabolic responses (11, 12), and induce significant endurance and strength adaptations (13). In previous studies, EMS application to the lower extremity muscles in patients depending on a wheelchair acutely increased cardiac output and V̇O_2_ (11, 12, 14). Nevertheless, these studies often featured small sample sizes or lacked adequate control conditions and randomization. Additionally, previous research often chose to initiate leg movement via a specific computer-controlled pattern using functional electrical stimulation. For this method, however, low metabolic efficiency was reported, leading to recommendations for alternative stimulation methods (15).

In terms of EMS frequencies, it is hypothesized that lower frequencies may induce lower fatigue, potentially extending time to exhaustion and allowing for longer training durations or higher volumes (16). Notably, higher increases in key metabolic transcript factors were reported after low-frequency stimulation at 5Hz compared with pulse-matched stimulation at 20Hz (17), suggesting higher adaptation potential at lower stimulation frequencies. Moreover, the simultaneous use of EMS at 4Hz on lower extremity muscles resulted in 39.7% (30.0) higher V̇O_2_ during submaximal handcycling in healthy young adults (18). How-ever, considering the substantially lower total muscle mass in the lower extremities of SCI patients (19), it is uncertain whether similar oxygen response levels can be achieved in this population. Even though WCB is not exclusively played by players with SCI, but rather by athletes with a variety of disabilities (20), similar lower acute oxygen responses are to be expected in wheelchair-dependent athletes overall.

Therefore, we aimed to elucidate whether these results of the aforementioned study (18) can be replicated in a similar study design in elite WCB athletes. We hypothesize that applying EMS to the lower extremities will acutely result in a significant increase in V̇O_2_, potentially influencing handcycling training for wheelchair sports athletes.

## METHODS

### Participants

Based on the data of a previously published pilot study (18), an a priori power analysis employing G*Power was conducted (Version 3.1.9.7, University of Kiel, Germany). Due to the lower to-be-stimulated muscle mass in the wheelchair population compared with abled-bodied participants, an effect size of half the magnitude as reported in the pilot study was used for sample size estimation (*α* = 0.05, study power (1-*β*-error) = 0.95, *r* = 0.35, effect size η_p_² = 0.43 [f = 0.25]), thus revealing a required sample size of *n* = 8 participants. Accounting for dropouts, *n* = 12 male wheelchair basketball players from the German national team were enrolled in this acute intervention trial (see Table I). The injury classifications of the players ranged from 1.0 to 4.5 with class 1.0 corresponding to the least mobility and class 4.5 corresponding to the most mobility based on the medical assessment in classification of athletes with disability (20). participants were instructed to avoid strenuous exercise 48 h prior to their study attendance. The study was approved by the ethical committee of the German Sport University Cologne (001/2020). All participants signed informed written consent prior to the start of the study.

**Table I:** Participants’ characteristics

Subject	Age (years)	Mass (kg)	Height (m)	Active (national team) (years)	Functional classification	Lesion/injury	Time since injury (years)
1	35	90.0	2.00	11 (7)	4.5	Knee injury	11
2	27	46.2	1.50	20 (8)	1.0	Spinal cord injury	22
3	20	61.2	1.70	10 (1)	2.5	AMC Type I	Birth
4	18	86.8	1.85	6 (1)	3.5	HSP	Birth
5	31	63.4	1.63	17 (3)	3.0	Spina bifida	Birth
6	22	90.3	1.86	10 (0.5)	4.5	Cerebral palsy	Birth
7	34	66.4	1.61	21 (12)	2.0	CRS	Birth
8	29	57.0	1.69	22 (7)	3.0	Spina bifida	Birth
9	22	103.6	1.98	8 (3)	3.5	Spina bifida	Birth
10	22	53.9	1.73	8 (1)	1.0	Spinal cord injury	15
11	24	56.3	1.59	18 (6)	2.0	Spinal stenosis	Birth
12	23	112.9	1.85	13 (4)	4.5	ECF	13

HSP: hereditary spastic paraplegia; AMC: arthrogryposis; CRS: caudal regression syndrome; ECF: epiphysiolysis capitis femoris.

### Study design

This acute intervention trial consisted of a single lab visit. After anthropometric assessment, all participants were briefly familiarized with EMS. For familiarization, at first, the EMS stimulation intensity corresponding to the individual maximal tolerable pain threshold (see Data acquisition) was determined and afterwards applied for a period of 2–3 min. Thereafter, participants completed the acute intervention protocol consisting of 4 x 5-min intervals interspersed with 3 min of passive rest. During these 4 x 5-min sequences, all participants completed 2 bouts of handcycling (HANDCYCLE) and 2 bouts of handcycling with concurrent electrical stimulation of the lower extremities (EMS_HANDCYCLE). The order of these 2 conditions was randomized for all participants.

### Data acquisition

During EMS_HANDCYCLE, low-frequency EMS (LF-EMS; impulse frequency: 4Hz, impulse width: 350 µs, continuous stimulation pattern) was applied to the lower limbs. For this, a pair of surface electrodes (MIHA BODYTEC II, miha bodytec GmbH, Gersthofen, Germany) were positioned each on the buttocks, the thighs, and the calves of the participants. Depending on the circumference of the hip, upper leg, and lower leg, different sizes of belt electrodes (size S, M, or L) were used to stimulate the glutei muscles (electrode length × electrode height: 13 cm × 10 cm), the hamstring and quadriceps muscles (20.5–60.5 cm × 4cm) and the triceps surae (20.5–32.5 cm × 4cm). During the familiarization phase prior to the acute intervention, the individual maximal tolerable pain threshold (IPT; maximal stimulation intensity that could be endured for the duration of the study) was determined. For this, the main controller of the EMS controlling device (MIHA BODYTEC II, miha bodytec GmbH, Gersthofen, Germany) was cranked up to its maximal intensity (100%, 120mA). Thereafter, by using the respective individual controller on the EMS controlling device, the IPT was determined for each pair of electrodes separately by beginning at the glutei muscles and continuing with the hamstring/quadriceps muscles and calves. As an orientation, a similar stimulation intensity was aimed for compared with the previously conducted pilot study (buttocks: 80.0 [22.7] mA, thighs: 94.5 [20.5] mA, calves: 77.5 [19.1] mA (18)). The stimulation intensity (buttocks: 69.5 [22.3] mA, thighs: 66.8 [20.0] mA, calves: 68.9 [31.5] mA) that ultimately reached the muscles cannot be precisely determined due to differences in the tissue resistance and structures (21).

Load during HANDCYCLE and EMS_HANDCYCLE consisted of handcycling performed on a medically verified handcycle ergometer (Dynamed Motion body 600, Stolzenberg GmbH, Erftstadt, Germany). During both HANDCYCLE and EMS_HANDCYCLE the load was set at 75% of the respective participant’s body mass. For all intervals, participants were instructed to reach and maintain a cranking cadence of 60 rpm with visual feedback provided by the corresponding software of the handcycling ergometer.

Breath-by-breath respiratory data were continuously recorded throughout the whole session using a validated spirometric system (Zan 600, KoKo GmbH, Oberthulba, Germany). Following the manufacturer’s recommendations, this spirometric system was calibrated prior to each test. Furthermore, the participant’s heart rate (HR) was recorded at 1Hz using a chest strap (H9, Polar Electro, Kempele, Finland). V̇O_2_, HR, total ventilation volume (VE), breathing frequency (BF), and carbon dioxide release (V̇CO_2_) were averaged over the last 3 min for each of the 4 x 5-min sequences. Additionally, by dividing V̇CO_2_ by V̇O_2_, the respiratory exchange ratio (RER) and by dividing V̇O_2_ by HR the oxygen pulse (O_2_ Pulse) were calculated. After each 5-min sequence, participants were asked to rate their perceived effort (RPE; CR-10) (22) and discomfort on a scale of 0 (no discomfort) to 10 (maximal discomfort) (23). Immediately prior and after each of the 4 x 5-min sequences, 20 µl capillary blood samples were obtained from the earlobe to determine the blood lactate concentration (Biosen C-Line; EKF Diagnostic Sales, Magdeburg, Germany). Subsequently, the difference between the pre- and post-value for each 5-min sequence was computed (ΔLactate).

For all further analyses, the respective values for the two bouts HANDCYCLE and EMS_HANDCYCLE were averaged, respectively.

### Statistical analysis

All data are presented as mean value and standard deviation (SD). All dependent variables were initially checked for normal distribution and variance homogeneity by combining visual inspection and employing Shapiro–Wilk tests. To examine “condition” differences (HANDCYCLE vs EMS_HANDCYCLE) repeated measures of variance (rANOVA) were separately conducted for the respective dependent variables (V̇O_2_, V̇CO_2_, VE, BF, RER, O_2_ pulse, ΔLactate, and HR). Effect sizes for rANOVA are provided as partial eta squared (η_p_^2^) with ≥ 0.01, ≥ 0.06, ≥ 0.14 indicating small, moderate, and large effects, respectively (24). Furthermore, for pairwise effect size comparison, standardized mean differences (SMDs) were calculated as the differences between the means divided by the pooled standard deviations (trivial: | SMD | < 0.2, small: 0.2 ≤ | SMD | < 0.5, moderate: 0.5 ≤ | SMD | < 0.8, large: | SMD | ≥ 0.8) (24). All statistical analyses were performed using R (version 4.2.0) in its integrated development environment RStudio (Version 2022.02.3+492, R Core Team, 2022; R Foundation for Statistical Computing, Vienna, Austria).

## RESULTS

**Fig. 1:**
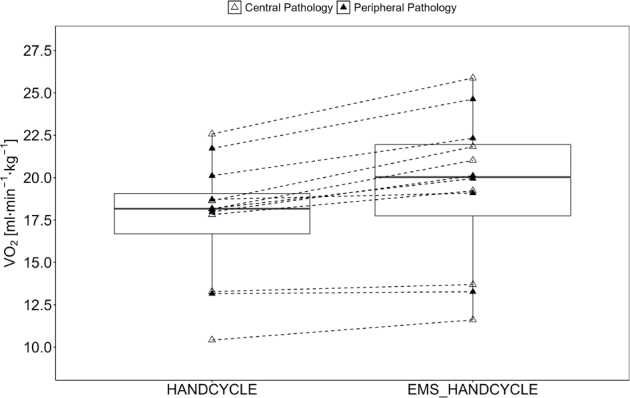
Acute oxygen uptake. Boxplot (Q1 to Q3, including median), and whiskers (showing minimum and maximum values) of oxygen uptake (V̇O_2_) during handcycling (HANDCYCLE) and handcycling with concurrent electrostimulation of the legs (EMS_HANDCYCLE). Individual values are presented as white triangles for participants with central pathology (i.e., spinal cord injury, hereditary spastic paraplegia, cerebral palsy, caudal regression syndrome, spinal stenosis) and black triangles for participants with peripheral pathology (i.e., knee injury, arthrogryposis Type I, epiphysiolysis capitis femoris, spina bifida). *P*-values of rANOVA and standardized mean difference (SMD) of pairwise comparison are indicated.

The rANOVA showed a statistically significant and large effect for V̇O_2_ (F[1, 11] = 24.10, *p* < 0.001, η_p_^2^ = 0.687) with higher values in EMS_HANDCYLE compared with HANDCYLE (17.60 [3.57] vs 19.23 [4.37] ml min^–1^ kg^–1^) (Fig. 1).

The rANOVA also revealed large and significant “condition” differences for V̇CO_2_ (F[1, 11] = 24.46, *p* < 0.001, η_p_^2^ = 0.690), VE (F[1, 11] = 20.56, *p* < 0.001, η_p_^2^ = 0.651), BF (F[1, 11] = 5.08, *p* = 0.046, η_p_^2^ = 0.316), RER (F[1, 11] = 4.92, *p* = 0.049, η_p_^2^ = 0.309), O_2_ pulse (F[1, 11] = 15.58, *p* = 0.002, η_p_^2^ = 0.586), HR (F[1, 11] = 5.67, *p* = 0.036, η_p_^2^ = 0.340) (Table II).

**Table II:** Comparison of performance parameters

Parameter	HANDCYCLE	EMS_HANDCYCLE	*p*-value	SMD
V̇CO_2_ [l min^-1^]	1.18 ±0.33	1.32 ± 0.39	< 0.001	0.388
VE [l min^-1^]	29.90 ±8.44	33.61 ±10.33	< 0.001	0.393
BF [min^-1^]	20.0 ±5.3	21.8 ±6.7	0.046	0.302
RER [–]	0.87 ±0.05	0.90 ±0.05	0.049	0.600
O_2_ Pulse [ml]	16.7 ±4.5	18.4 ±5.2	0.002	0.351
HR [min^-1^]	101.0 ±12.1	103.8 ±11.0	0.036	0.243

Comparison between handcycling (HANDCYCLE) and handcycling with concurrent electromyostimulation of the legs (EMS_HANDCYCLE). Standardized mean differences (SMD) and *p*-value of rANOVA are also provided.

**Fig. 2:**
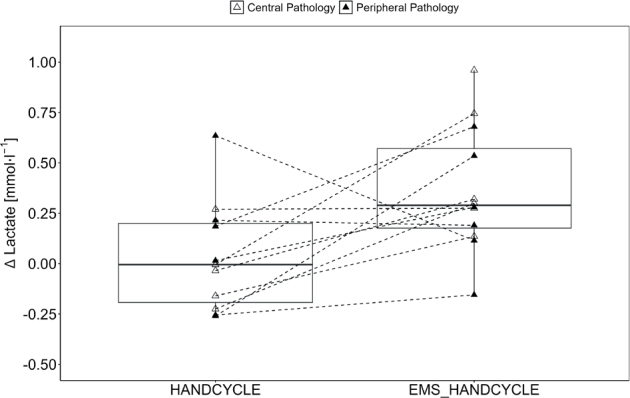
Lactate accumulation. Boxplot (Q1 to Q3, including median), and whiskers (showing minimum and maximum values) of POST-PRE differences in blood lactate level (ΔLactate) during handcycling (HANDCYCLE) and handcycling with concurrent electrostimulation of the legs (EMS_HANDCYCLE). Individual values are presented as white triangles for participants with central pathology (i.e., spinal cord injury, hereditary spastic paraplegia, cerebral palsy, caudal regression syndrome, spinal stenosis) and black triangles for participants with peripheral pathology (i.e., knee injury, arthrogryposis Type I, epiphysiolysis capitis femoris, spina bifida). *P*-values of rANOVA and standardized mean difference (SMD) of pairwise comparison are indicated.

Furthermore, a statistically significant and large effect for ΔLactate (F[1, 10] = 5.84, *p* = 0.036, η_p_^2^ = 0.369) with higher values in EMS_HANDCYLE compared with HANDCYLE (0.31 [0.26] vs 0.04 [0.28 mmol l^–1^]) were found as absolute values increased from 1.48 (0.24) mmol l^–1^ to 1.85 (0.42) mmol l^–1^ during EMS_HANDCYCLE and from 1.43 (0.22) mmol l^–1^ to 1.54 (0.42) mmol l^–1^ during HANDCYCLE (Fig. 2).

Rating of perceived effort did not differ between EMS_HANDCYLE and HANDCYLE (F[1, 11] = 1.221, *p* = 0.293, η_p_^2^ = 0.100; 2.65 (0.90) vs 2.92 (0.85), SMD = 0.311). However, discomfort was rated statistically significant higher during EMS_HANDCYLE compared with HANDCYLE (F[1, 11] = 15.17, *p* = 0.002, η_p_^2^ = 0.580; 1.44 [1.28] vs 3.94 [ 2.14]., SMD = 1.419).

## DISCUSSION

This controlled crossover study aimed at elucidating the acute effect of concurrent low-frequency electromyostimulation (LF-EMS) of the lower extremities on oxygen uptake during submaximal handcycling in elite wheelchair basketball athletes. The main findings of our study indicate that the application of LF-EMS to the lower limbs during handcycling acutely increases the oxygen demand and other spirometric parameters such as carbon dioxide release, total ventilation volume, breathing frequency, respiratory exchange ratio, oxygen pulse, and the heart rate significantly. Furthermore, during handcycling with concurrent LF-EMS a significantly higher onset of blood lactate was observed compared with handcycling without electrical stimulation. Interestingly, perceived effort did not differ between the two conditions, even though discomfort was rated higher during handcycling with concurrent electrical stimulation.

In the present study, we found that the concurrent application of LF-EMS to the lower extremities during submaximal handcycling leads to an acutely increases the oxygen demand by 8.9% (5.8) (1.34 [0.34] l min^–1^ to 1.47 (0.39) l min^–1^) compared with submaximal handcycling without complementary LF-EMS. These increases are noticeably lower compared with the results of Rappelt and colleagues (18). In this study, concurrent LF-EMS with the same stimulation pattern as in the present study was applied to the lower extremities during submaximal handcycling (50% bodyweight as resistance) and led to acute increases in V̇O_2_ by 39.7% (30.0) (0.97 [0.21] l min^–1^ to 1.36 [0.44] l min^–1^) compared with handcycling without additional EMS stimulation. This study, however, was performed in healthy subjects at a higher stimulation intensity than in the present study (buttocks 80.0 [22.7] mA vs 69.5 [22.3] mA; thighs: 94.5 [20.5] mA vs 66.8 [20.0] mA; calves: 77.5 [19.1] mA vs 68.9 [31.5] mA). As oxygen consumption seems to increase with increasing electrical stimulation intensity (25) and to depend on how much muscle mass is involved in a particular exercise (5), it seems plausible that in the present study in a wheelchair population, with most likely lower muscle mass in the lower extremities (19) and stimulation at a lower intensity, we found smaller increases in oxygen demand. Our results are, however, in line with results reported by Thomas and colleagues (26): in that study, the application of concurrent electrical stimulation of the leg muscles during submaximal hand cranking exercise at 50% of V̇O_2_max led to significant increases in V̇O_2_ from 0.75 (0.11) to 0.83 (0.10) l min^–1^ in 6 participants with SCI. Similarly, also in SCI patients, increases in V̇O_2_ of up to 35% were found during arm-cranking exercises at maximum working intensity, when additional functional electrical stimulation of the lower limbs was induced (27, 28). However, in these studies, by employing functional electrical stimulation of the legs, a cycling movement on an ergometer was induced, whereas in the present study the legs were at rest. Nevertheless, it seems that, regardless of the stimulation method, concurrent application of EMS on the lower extremities acutely increases the oxygen demand during handcycling across various working intensities. The magnitude of the increase in V̇O_2_, however, may be dependent on the stimulated muscle mass and the applied stimulation intensity.

During EMS training, motor units are recruited in a nonselective pattern with both slow-twitch and fast-twitch muscle fibres being innervated simultaneously (29). However, as fast-twitch fibres seem to be predominantly allocated at the surface, while slow-twitch fibres are more frequent in deeper regions of the muscle (30), fast-twitch fibres closer to the EMS electrodes placed on the skin are thus more likely to be stimulated. As these fast-twitch fibres produce lactate at a higher rate compared with slow-twitch muscle fibres (31), this leads to an onset of blood lactate. This effect might be even more pronounced in wheelchair-dependent populations, as in the years following a SCI, due to immobilization, a progressive decrease in the proportion of slow-twitch fibres with a simultaneous rise of almost exclusively fast-twitch fibre expression has been reported (32). However, the increase in blood lactate during handcycling with concurrent LF-EMS was lower in the present study (+0.31 [0.26] mmol l^–1^) compared with the pilot study conducted in able-bodied individuals (+0.86 [0.63] mmol l^–1^) (18). The higher values in the pilot study might be explained by (*i*) the most likely larger muscle mass in the lower extremities of the able-bodied participants, (*ii*) the higher stimulation intensity (buttocks 80.0 [22.7] mA vs 69.5 [22.3] mA; thighs: 94.5 [20.5] mA vs 66.8 [20.0] mA; calves: 77.5 [19.1] mA vs 68.9 [31.5] mA) and the fact that (*iii*) only 2 athletes in the present study had an SCI, so that the shift towards exclusively type-II fibres may be less pronounced in the sampled population. Nevertheless, the higher metabolic load induced by an increased intramuscular concentration of lactate, which in turn is associated with long-term mitochondrial adaptations (33), has been suggested as a possible underlying mechanism for long-term increases in aerobic capacity induced by the application of LF-EMS (34, 35). Moreover, at least in acute settings, EMS was found to increase the venous blood flow (8, 9), which is associated with an increased end-diastolic volume and thus directly linked to (maximal) oxygen uptake (7). This is also a possible explanation for our findings of a higher O_2_ pulse during handcycling with concurrent LF-EMS. Additionally, in terms of peripheral factors affecting the oxygen uptake, after 18 weeks of strength training with superimposed whole-body EMS, improvements in the deformability of red blood cells was reported (36). Thus, it is not entirely clear which factors will potentially lead to long-term adaptations in (maximal) oxygen uptake induced by EMS. Nevertheless, the positive effect of EMS on cardiovascular adaptations has been reported several times, at least in able-bodied individuals (34, 37). Similarly, in SCI patients, concurrent electrical stimulation of the lower limbs during handcycling led to significant increases in V̇O_2_max (14). However, this study consisted of 3 successive training blocks in non-randomized order without a control group. Therefore, further randomized control trials are needed to (*i*) clarify the extent to which the concurrent electrical stimulation of the lower extremities during handcycling affects the cardiopulmonary capacity in wheelchair-dependent populations, and (*ii*) which underlying physiological adaptions lead to these adaptations.

Another tangible benefit of increasing blood flow to the lower limbs may lie in reducing the occurrence of pressure ulcers in wheelchair-dependent populations (40) – one of the most common complications and most frequent cause of (re-)hospitalization in SCI (38) and spina bifida patients (39). At least in SCI patients, with the acute application of electrical stimulation of the gastrocnemius muscle (10 sets of 1 min stimulation/1 min rest; 8Hz, 400µs at 46.6 [12.3] mA), a mean increase of 13.0% (4.9) in blood flow of the popliteal artery of the calf was reported (41).

A limitation to be addressed is the heterogeneity of the included participants (i.e., different pathologies, muscle mass in the lower extremities). This is of particular importance as individual differences in the tissue structure and tissue resistance have an impact on the stimulation intensity that ultimately reaches the muscles (21). Nevertheless, underlying molecular responses to electrical muscle stimulation are considered intact even after muscular denervation (42), thus allowing for muscular adaptations; especially when initiated early after a traumatic spinal cord injury (43), but even years after the injury occurred (44). Moreover, we focused only on acute effects at comparable low exercise intensities. While our results are promising, scientific evidence regarding the long-term training effects of concurrent low-frequency electromyostimulation on aerobic capacity remains limited. Nevertheless, even in our heterogeneous sample (i.e., consisting of participants with both central and peripheral impairments), the overall acute effects are meaningful in their size, homogeneous in their direction and no adverse events were reported by the participants. Thus, further longitudinal randomized control trials with a special emphasis on the underlying physiological responses are necessary to disentangle the relationship of a potential EMS-induced increase in lower extremity muscle mass, fibre type distribution, and its effect on health and performance parameters in wheelchair athletes.

In conclusion, the concurrent application of low-frequency electromyostimulation during handcycling significantly increases the acute oxygen demand. However, further research is necessary to determine whether similar increases in the acute oxygen demand are also present when applying concurrent EMS during handcycling at higher intensities. Furthermore, while the concurrent electrical stimulation may help increasing the acute haemodynamic response, long-term adaptations in V̇O_2_max and possible underlying physiological mechanisms should be evaluated in future longitudinal randomized controlled trials in wheelchair populations.
